# C8 Health, a Platform for the Implementation of Best Practices: Survey-Based Usability Study

**DOI:** 10.2196/83547

**Published:** 2026-06-05

**Authors:** Faria Nisar, Nicolas Mario D Alessandro, Jessica Suratkal, Ido Zamberg, Samantha Elen Pope, Ali Ali, Luis Etienne Tollinche

**Affiliations:** 1 Department of Anesthesiology and Pain Management, School of Medicine, The MetroHealth System, 2500 MetroHealth Drive, Cleveland, OH, 44109-1998, United States, 1 2167782247; 2 Case Western University School of Medicine, Cleveland, OH, United States; 3 School of Education, Johns Hopkins University, Baltimore, MD, United States; 4 Case Comprehensive Cancer Center, Case Western Reserve University, Cleveland, OH, United States; 5 Outcomes Research Consortium, Cleveland, OH, United States

**Keywords:** C8, mobile health, mHealth, mHealth App Usability Questionnaire, MAUQ, usability, quality initiative, anesthesiology

## Abstract

**Background:**

Mobile health (mHealth) apps are increasingly integrated into clinical workflows to support decision-making and adherence to best practices. Usability is a critical determinant of adoption, engagement, and long-term use.

**Objective:**

This study aimed to evaluate the usability of the C8 Health platform deployed in the Department of Anesthesiology at MetroHealth in 2024.

**Methods:**

A quality improvement initiative was conducted using the mHealth App Usability Questionnaire (MAUQ). A total of 142 anesthesiology clinicians participated by completing the questionnaire. This study was reported in accordance with the SQUIRE (Standards for Quality Improvement Reporting Excellence) guidelines for quality improvement reporting.

**Results:**

The overall MAUQ scores indicated high usability, with mean scores greater than 5.5 across core items (overall mean MAUQ score 5.73, SD 0.81). These findings suggest strong user satisfaction and positive engagement with the C8 Health platform among anesthesiology clinicians.

**Conclusions:**

The C8 Health platform demonstrated high usability in an anesthesiology setting. These results support its integration as a clinical decision support tool to enhance workflow efficiency and adherence to best practices.

## Introduction

Mobile health (mHealth) technologies have become integral to modern health care, offering “low-cost and high accessibility” solutions that empower clinicians and patients alike and help streamline clinical workflows [1,2]. However, a significant barrier to adoption lies in usability. Poor app design, data entry burden, and frustrating interfaces often cause users to abandon these tools. To address this, the mHealth App Usability Questionnaire (MAUQ) was developed as a validated, reliable instrument tailored specifically for evaluating usability in health care apps, covering ease of use, interface satisfaction, and perceived usefulness [3].

In June 2024, the MetroHealth System introduced C8 Health, an mHealth app designed to help health care institutions implement their best practices effectively through the centralization of clinical protocols, checklists, and workflows for care teams. Though early feedback has been promising [4-6], systematic usability evaluation among end users has not yet been conducted.

This study aimed to fill that gap by assessing C8 Health’s usability among MetroHealth anesthesia clinicians using the MAUQ. Evaluating usability at this stage is critical not only to refine the app’s user interface and functional workflow but also to understand how such tools might support scalable, protocol-driven perioperative care [7]. The results are expected to guide both iterative improvements to C8 Health and inform frameworks for introducing similar mHealth solutions in hospital settings.

## Methods

### Study Design and Setting

This was a cross-sectional survey study conducted in the Department of Anesthesiology at the MetroHealth System, a tertiary academic medical center in Cleveland, Ohio, United States. The study evaluated the usability of C8 Health, a mHealth app introduced in June 2024 to support clinical workflow efficiency and adherence to best practices. This study was reported in accordance with the SQUIRE (Standards for Quality Improvement Reporting Excellence) guidelines for quality improvement reporting.

### Participants and Recruitment

**Figure 1.**
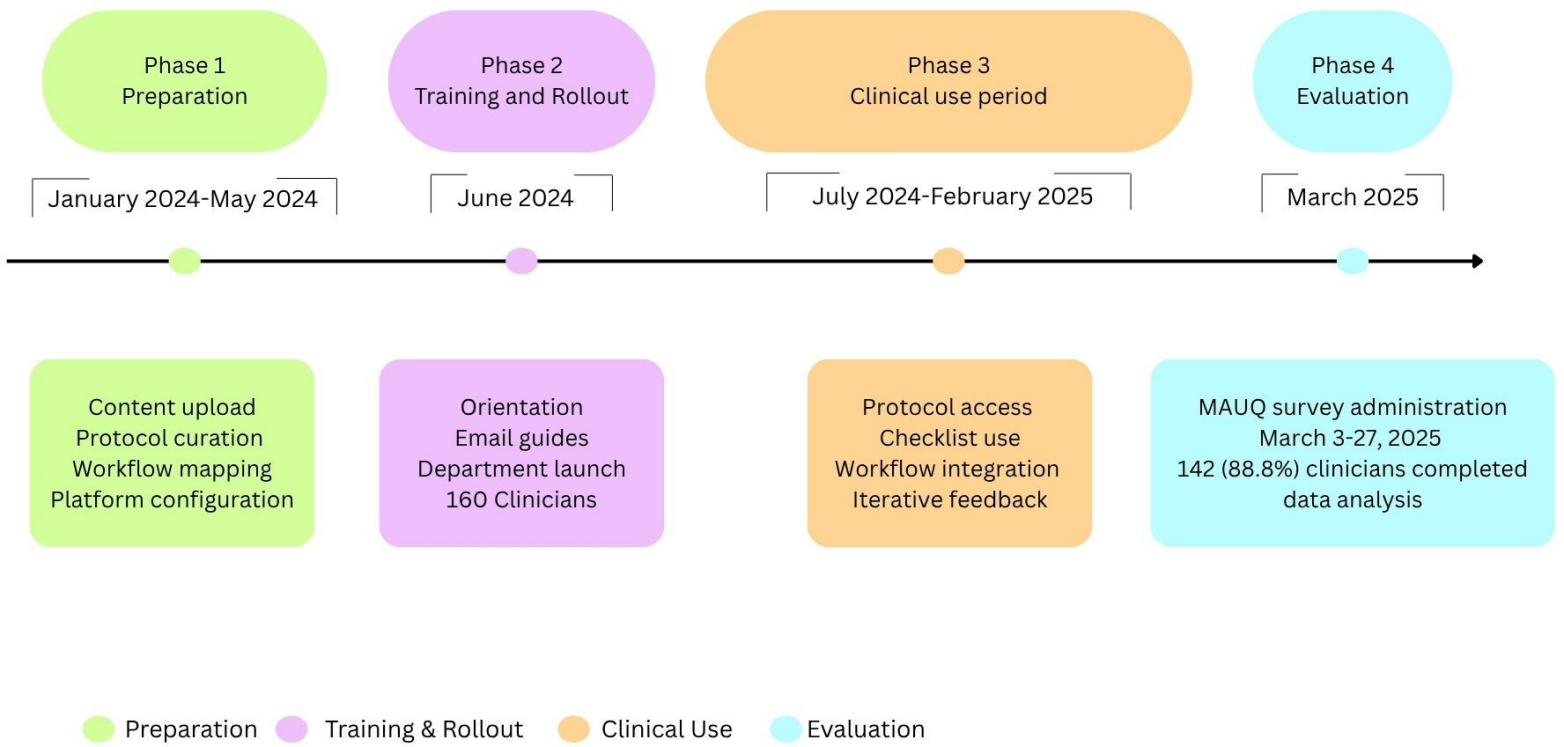
Implementation timeline of the C8 Health platform in the Department of Anesthesiology and Pain Management, the MetroHealth System, Cleveland, Ohio, United States. MAUQ: mHealth App Usability Questionnaire.

All anesthesiology clinicians employed at the MetroHealth System during the study period were invited to participate. Inclusion criteria included active clinical practice in the department and familiarity with the C8 Health platform. Participation was voluntary and anonymous. Recruitment was conducted via institutional email invitations, and nonresponders received 2 to 3 reminder emails per week from research faculty to maximize response rates (Figure 1).

### Data Collection

The survey was administered online using SurveyMonkey (SurveyMonkey Inc) between March 3 and March 27, 2025. Survey responses were deidentified to preserve confidentiality. No personally identifiable information was collected.

### Measures

Usability was assessed using the validated MAUQ, which evaluates 3 domains: ease of use, system information arrangement, and usefulness in health care delivery. The questionnaire uses a 7-point Likert scale, with higher scores indicating greater usability. Open-ended questions were also included to capture qualitative feedback regarding functionality, interface design, and overall user experience.

### Outcomes

The primary outcome was the overall usability score derived from the MAUQ. Secondary outcomes included subscale scores for usability domains and thematic analysis of open-ended responses to identify strengths and areas for improvement.

### Statistical Analysis

Descriptive statistics were used to summarize the survey responses. Continuous variables (eg, MAUQ scores) were reported as means with SDs. Subscale analyses were performed to compare usability domains. Open-ended comments were analyzed thematically to identify recurring usability issues and user recommendations.

### Ethical Considerations

The study received institutional review board exemption (STUDY00000729) from the MetroHealth System, as it was classified as a quality improvement initiative involving minimal risk to participants. As the study was granted an institutional review board exemption, informed consent was waived. All data were handled in accordance with institutional privacy and confidentiality standards, and no compensation was provided to participants.

## Results

The survey was distributed to all 160 clinicians, including faculty physicians, residents, and departmental staff, yielding a response rate of 88.8% (n=142).

Overall, results showed high usability ratings. On a 7-point Likert scale, the app received weighted averages above 5.5 for most core items. Clinicians strongly agreed that the app was easy to use (122/142, 86%; mean 5.73), easy to learn (124/142, 87%; mean 5.78), and supported consistent navigation (122/142, 86%; mean 5.70). Additionally, participants (112/142, 79%) valued the design and were able to access all key functions (mean 5.50). Because of the straightforward interface, 84% (119/142) of respondents felt the time required to use the app was appropriate.

Surveyed clinicians also found the information distributed by C8 Health beneficial, as 79% (112/142) of users appreciated the app’s serviceability. The app was perceived as valuable in practice, with strong agreement that it improved health care service access (mean 5.66) and supported effective patient health management (mean 5.54, SD).

Although largely positive, the survey highlighted areas for improvement. Offline utility received a weighted average of 4.95, with only 58% (82/142) of users finding this component satisfactory. Additionally, error recovery received a lower average of 5.28 (SD), yet 67% (95/142) of participants felt satisfied with this function.

**Figure 2.**
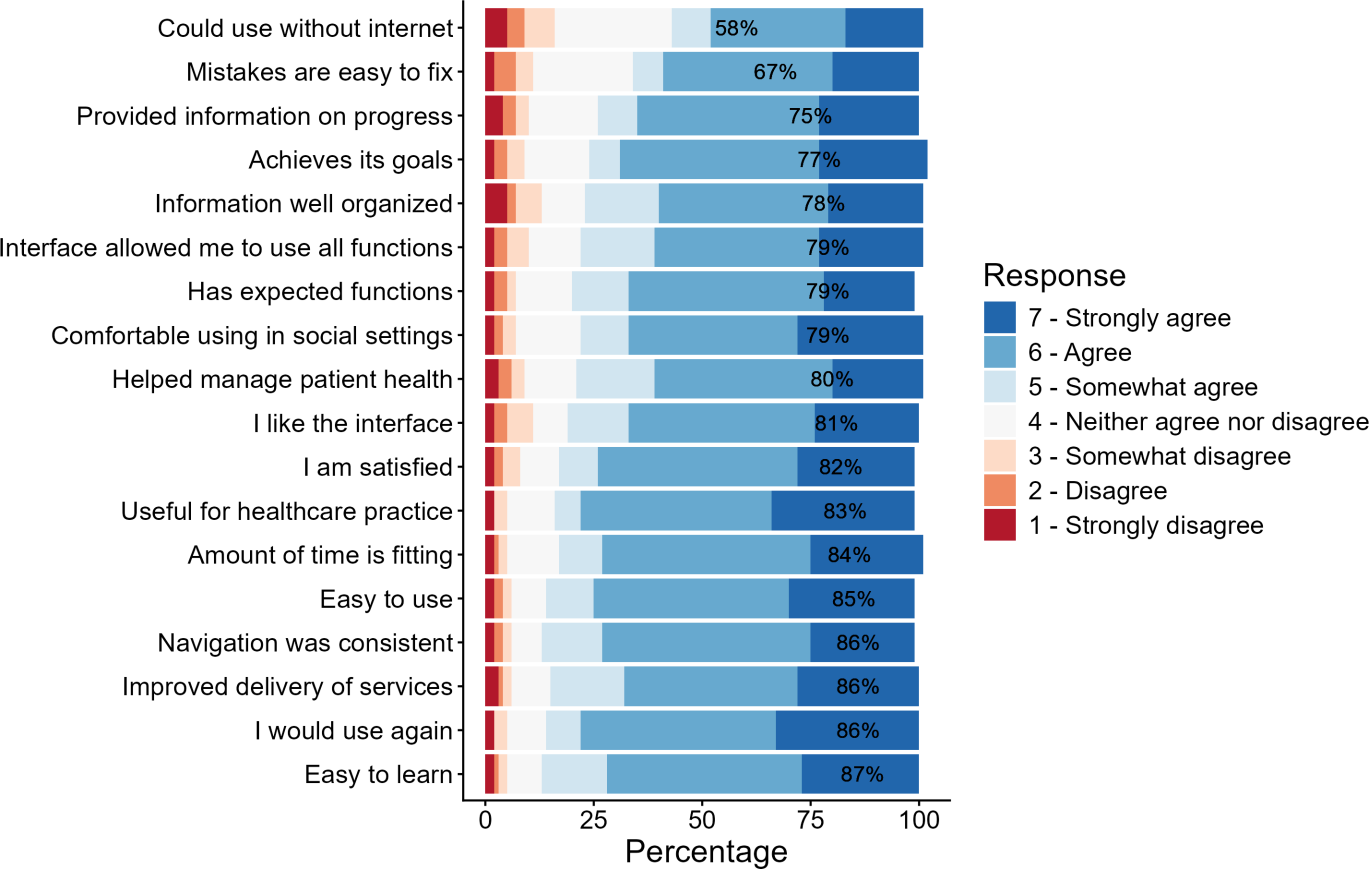
mHealth App Usability Questionnaire survey results with percentages representing the summation of “agree” and “strongly agree” responses.

However, overall satisfaction remained high (mean 5.72), affirming the app’s acceptance and perceived benefit in a clinical setting. Respondents strongly agreed that they would use C8 Health again (mean 5.87). These findings (Figure 2) validate the C8 Health app’s design and implementation as a useful, user-friendly digital tool for anesthesiology professionals. Ongoing iterative improvements, particularly focused on offline functionality and error handling, are recommended to maximize utility and engagement.

## Discussion

### Principal Findings

In this usability study, of the 160 clinicians from the Department of Anesthesiology at the MetroHealth System, 142 (88.8%) completed the MAUQ questionnaire to assess C8 Health, an mHealth app that centralizes clinical protocols and checklists to enhance clinical workflow and adherence to best practices. The MAUQ is a validated tool to assess the usability of the mHealth platform targeted for health care providers. Overall, the scores showed high usability and satisfaction, with weighted averages above 5.5 on a 7-point Likert scale for key aspects such as ease of use, accessibility of functions, design appreciation, and perceived value in practice.

Beyond global usability ratings, individual MAUQ items provide insight into how C8 Health supported clinical practice. A total of 86% (122/142) of respondents agreed or strongly agreed that the platform improved the delivery of health care services, and 83% (118/142) found it useful for their health care practice, reflecting its integration into day-to-day clinical workflow. Notably, 80% (114/142) agreed that it helped them manage patient health, suggesting that clinicians perceived the platform as more than an informational repository but rather as an active support tool in patient care decisions. The high agreement on information organization (111/142, 78%) and availability of expected functions (112/142, 79%) further suggested that clinicians could reliably locate protocols and checklists when needed at the point of care, a key requirement in time-sensitive perioperative environments such as the operating room.

### About C8 Health

C8 Health is a platform for the implementation of best practices that centralizes medical knowledge to streamline health care operations and improve patient care. It disseminates essential information such as clinical protocols, policies, procedures, guidelines, checklists, and workflows, giving care teams fast access to standardized, evidence-based resources [7]. Apps such as C8 Health have the potential to offer benefits in health care by improving access to standardized clinical knowledge with the goal of reducing care variability and informing clinical decision-making. By unifying resources and enabling seamless knowledge sharing across departments and organizations, these tools may support more consistent, accurate, and timely care delivery. Whether this translates to direct improvements in patient outcomes, including reductions in medical errors or mortality rates, remains to be established through controlled outcome studies.

### Current State of Best Practices Implementation in Health Care

Adherence to established clinical protocols significantly enhances patient outcomes by reducing complications, shortening hospital stays, and lowering health care costs. For instance, following the Enhanced Recovery After Surgery (ERAS) protocol in breast reconstruction has been shown to decrease postoperative complications and reduce hospital length of stay [8]. Similarly, compliance with anesthesia-related ERAS measures, such as multimodal analgesia and opioid-sparing strategies, has been associated with significantly shorter hospital stays in colorectal and gynecologic surgeries [9,10]. Furthermore, the use of ventilators and infection prevention bundles in intensive care unit settings has been shown to decrease mortality and ventilator-associated pneumonia rates when properly implemented [11,12]. Nonadherence to anesthesia guidelines in pain interventions has also been linked to severe complications, emphasizing the risks of deviation [13]. Collectively, these findings reinforce that rigorous protocol compliance in surgical and anesthetic care settings leads to safer patient outcomes, with patients subsequently facing fewer complications and lower out-of-pocket expenses related to preventable hospitalizations or errors.

Current adherence to medical protocols such as the ERAS protocol remains suboptimal despite widespread implementation. A recent large-scale study found that the actual compliance rate with the ERAS protocol was only 32.3%, with a potential compliance rate of 53.2% if patients without postoperative medical issues were included [14]. These findings are consistent with broader literature, which highlights that adherence to medical protocols is often challenged by clinical complexity and organizational factors [15]. Thus, while protocols such as ERAS have proven benefits, real-world adherence is influenced by a complex interplay of patient, health care provider, and system-level factors [14,15].

Systems for implementation of best practices, such as C8 Health, have demonstrated the potential to improve adherence to medical protocols by providing clinicians with timely, evidence-based recommendations and reminders integrated into clinical workflows. Multiple studies indicate that similar interventions are associated with small to moderate improvements in the delivery of guideline-recommended care, with average absolute increases in process adherence of approximately 5.8% and best-case improvements reaching up to 8.5% [16]. Features such as treatment recommendations and automated flagging of best-practice alerts are particularly effective in supporting guideline adherence and quality assurance in patient care [17]. These systems can also enhance diagnostic accuracy, support monitoring, and facilitate preventive screening, all of which contribute to better protocol compliance [17]. Overall, while the extent of improvement varies, systems for implementation of best practices consistently contribute to better adherence to clinical protocols across diverse health care settings, indicating a substantial functional role of apps such as C8 Health in improving patient outcomes [16-18].

From the health care provider’s perspective, these digital tools may enhance the experience of delivering care by potentially reducing cognitive burden, supporting confidence in clinical decisions, and encouraging a culture of collaboration and transparency. Features such as natural language search, real-time updates, and integrated authoring tools help clinicians spend less time searching for information and more time focused on patient care. This contributes to reduced burnout and greater job satisfaction, both critical factors in an era marked by workforce shortages and growing demands on health care professionals [19].

### Assessing C8 Health

Considering the benefits of apps such as C8 Health to patients, health care providers, and health care institutions, one of the first steps in determining the feasibility of implementing this app is assessing usability. Using the MAUQ, the C8 Health app received a high overall MAUQ score among anesthesiologists at MetroHealth, with most core items receiving >5.5 on a 7-point Likert scale. Overall satisfaction with the app was high, with users agreeing that the app was easy to use with an agreeable interface. Notably, the app was perceived as valuable in practice, as users agreed that it improved health care service access and supported effective patient health management. These positive findings have important future implications: as the app was well perceived by anesthesiologists, broader adoption and integration into clinical workflows are increasingly possible, potentially supporting streamlined communication and increased efficiency, though improvements in patient outcomes remain hypothetical at this stage and require validation through prospective studies with objective outcome measures across health care institutions.

### Strengths and Limitations

Strengths of this study include that usability was assessed using the MAUQ, a validated and widely used instrument in mHealth research. The use of this tool increases both the reliability of our measurements and the comparability of findings with those from other studies in the field. The evaluation was conducted solely at a single postimplementation time point. The absence of preimplementation data precludes direct before-and-after comparisons of workflow efficiency, protocol adherence, or platform use, which would have provided stronger causal evidence. Furthermore, although voluntary participation in the questionnaire introduces the possibility of selection bias, this risk is mitigated by the exceptionally high response rate (142/160, 88.8%), which strengthens confidence in the representativeness of the data.

This study did not collect objective operational or clinical outcome data (eg, protocol adherence rates, time-to-information retrieval, or patient-level outcomes). Consequently, the improvements in workflow efficiency and care quality discussed herein are based on user perception rather than measured performance indicators.

One significant limitation of this study is that the evaluation was conducted at a single center and lacked a control group, both of which may restrict the generalizability of the findings. In addition, limitations of the C8 Health platform itself were identified. These include limited offline functionality, suboptimal error recovery, and a lack of integration with existing electronic health record systems. Such constraints may reduce workflow efficiency and continuity of care in practical settings. However, these issues represent identifiable opportunities for improvement to be addressed in future system development.

### Future Directions

In future research, quantifying improvements in patient care and institutional productivity following implementation of the app may assist in valuing the app in the context of outcomes and efficiency metrics. There is also a need for multicenter and longitudinal studies to assess sustained use. Future research should explore the integration of C8 Health with electronic health record systems to create a more comprehensive clinical decision support ecosystem. This integration could enable automated protocol selection based on patient characteristics, streamlined documentation workflows, and enhanced tracking of clinical outcomes. Future studies should also incorporate prospective pre-post designs with baseline data collection before implementation to better isolate the platform’s effect on clinical workflow and protocol adherence.

Overall, the strong usability and perceived value of the C8 Health app pave the way for it to become a transformative tool in perioperative care, setting a new standard for technology-driven support in anesthesiology and beyond.

### Conclusions

These findings indicate that the C8 platform demonstrates strong perceived usability and holds significant promise for supporting perioperative clinical workflows, as reported by end users. Ongoing development based on user feedback may further improve its effectiveness and extend its applicability to other clinical areas.

## Abbreviations

ERAS: Enhanced Recovery After Surgery
MAUQ: mHealth App Usability Questionnaire
mHealth: mobile health
SQUIRE: Standards for Quality Improvement Reporting Excellence
